# Global View of HIV Prevalence in Prisons: A Systematic Review and Meta-Analysis

**Published:** 2019-02

**Authors:** Mehdi SAYYAH, Fakher RAHIM, Gholam Abbas KAYEDANI, Kiarash SHIRBANDI, Amal SAKI-MALEHI

**Affiliations:** 1. Education Development Center (EDC), Ahvaz Jundishapur University of Medical Sciences, Ahvaz, Iran; 2. Research Center of Thalassemia & Hemoglobinopathy, Health Research Institute, Ahvaz Jundishapur University of Medical Sciences, Ahvaz, Iran; 3. Health Research Institute, Infectious and Tropical Diseases Research Center, Ahvaz Jundishapur University of Medical Sciences, Ahvaz, Iran; 4. Department of Virology, School of Medicine, Ahvaz Jundishapur University of Medical Sciences, Ahvaz, Iran; 5. Systematic Review and Meta-Analysis Expert Group (SRMEG), Universal Scientific Education and Research Network (USERN), Ahvaz, Iran; 6. Department of Biostatistics and Epidemiology, School of Health, Ahvaz Jundishapur University of Medical Sciences, Ahvaz, Iran

**Keywords:** HIV, Global, Systematic review, Meta-analysis, Prevalence, Prisoners

## Abstract

**Background:**

We aimed to estimate the global prevalence of HIV, as well as cross-countries comparison in people who are in prison.

**Methods:**

We systematically assessed published studies reporting HIV prevalence among prisoners in the world. We searched international datasets banks, including PubMed, SCOPUS, Cumulative Index to Nursing and ISI web of science along with local databases and included original articles reporting data on the prevalence of HIV from 1980 to 2017.

**Results:**

We included 72 studies that reported HIV prevalence for 2,275,930 adult male and female prisoners. The pooled estimate of HIV prevalence was 3.4% (95% CI 3.2%–3.6%); however, the prevalence of HIV across individual studies varied considerably (ranging from 0 in Bosnia and Herzegovina to More than 20% in Iran, Zambia, Spain) and statistical heterogeneity was substantial (I2=0.99, Q=121; *P*<0.0001). The prevalence of HIV among prisoners in the continents Asia, Africa, North America and Europe was estimated as 3.0% (95% CI 3.3%–4.3 %), 6% (95% CI −0.0%–2.0%), 4% (95% CI 3.0%–4.0%), 5.0% (95% CI 0.0%–11%), respectively.

**Conclusion:**

Protecting prisoners’ health protects general public health. Successful HIV preventive measures in prisons include provision of HIV education and information; clean needles and syringes; drug treatment; and condoms. Governments have a moral and ethical obligation to prevent the spread of HIV/AIDS in prisons and to provide compassionate care, treatment and support for those infected.

## Introduction

HIV/AIDS is a global problem that presents in different age groups and races, not only in homosexuals but in both sexes (1). Over the past few years, the issue of controlling high-risk diseases has been pursued among the prisoners to the detriment, and there are many efforts have been made since the prison environment is closed and the conditions for the transmission of contagious diseases are very much in place (2). HIV prevalence among prisoners has been reported to vary between different countries from 0%–2% in Australia to 2% in America, 11% in Latin American countries, 10% in the Middle East, and 20% in African countries (3). Despite the fact that addicts and drug offenders in prisons account for considerable part of the prison population, the number of people infected with HIV is higher, but the pace of its expansion is increasing (4).

“Such measurement also enables direct comparison of different HIV metrics, emphasizing the specific needs of each geographic region and allowing for a more targeted response to the epidemic” (5). Authorities should put urgent measures to prevent the transmission of HIV at the national level in the country’s agenda and plans because it will be too late tomorrow (5). We aimed to estimate the global prevalence of HIV, as well as cross-countries comparison in people who are in prison.

## Methods

### Search strategy

We performed a comprehensive search through international indexing databases, including PubMed, Scopus, Cochrane Library, PsycINFO, CINAHL, ISI Web of Science, Science Direct from inception, and Embase; moreover, local databases include SID (Scientific Information database, Magiran, and IRANDOC, were searched using both Persian and English keywords. The search was conducted from 1980 to 2017 with no language limit. Searched was performed using the following keywords: (HIV or Human Immuno-deficiency Virus or AIDS or Acquired Immune Deficiency Syndrome) and (prison or Prisoner) and (epidemiology or prevalence or incidence). In addition, to find more eligible studies the reference lists of relevant publications were manually searched. Studies that fulfilled defined criteria, including observational studies (prospective cohort, retrospective cohort, case-control, or cross-sectional) and reporting the prevalence of HIV among prisoners, which full-text was accessible, were considered in the meta-analysis. Other article types include reviews (narrative or systematic), commentaries, letters to the editor, case series or case reports, and pooled analyses of original data were excluded.

### Data extraction

Data were collected using a data extraction form, including first author name, publication year, location, study design, sample size, demographic characteristics such as age and sex, and criteria for enrolling. Two authors (F.R. and K.SH.) separately extracted the information of interest from studies. We contacted the authors of the eligible articles for missing data, if necessary.

### Statistical analysis

Heterogeneity was tested using both *I*^*2*^ statistic and Chi-square test. *I*^2^>50% or *P*<0.05 were considered to exhibit significant heterogeneity. Furthermore, funnel plot and Egger’s regression test was implemented to assess publication bias. Stata 13 was used to provide pooled estimations, with corresponding 95% CI and plots.

## Results

**Fig. 1:**
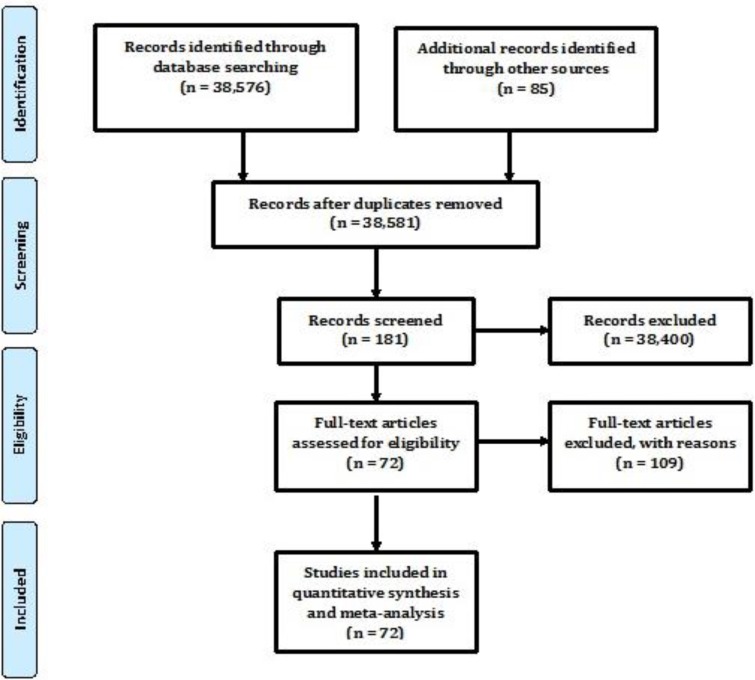
Study selection process, systematic review of HIV prevalence among prisoners

We found 3851 potentially relevant studies, of which 181 met our criteria and 72 articles (Fig. 1) (6–83), were included. Seventy-two selected studies enrolled 2,275,930 adult male and female prisoners. Of them, 32 were from Asia, 31 from North America, 5 from Europe, and 4 from Africa. The majority of included articles (n=64) were cross-sectional studies that assessed the prevalence of HIV among prisoners. Utmost included articles stated little to no information on possible factors accompanying with HIV prevalence. The results of our quality assessment alongside the evaluation criteria were summarized, that showed in most of the items more than 65% of criteria were fulfilled.

**Fig. 2:**
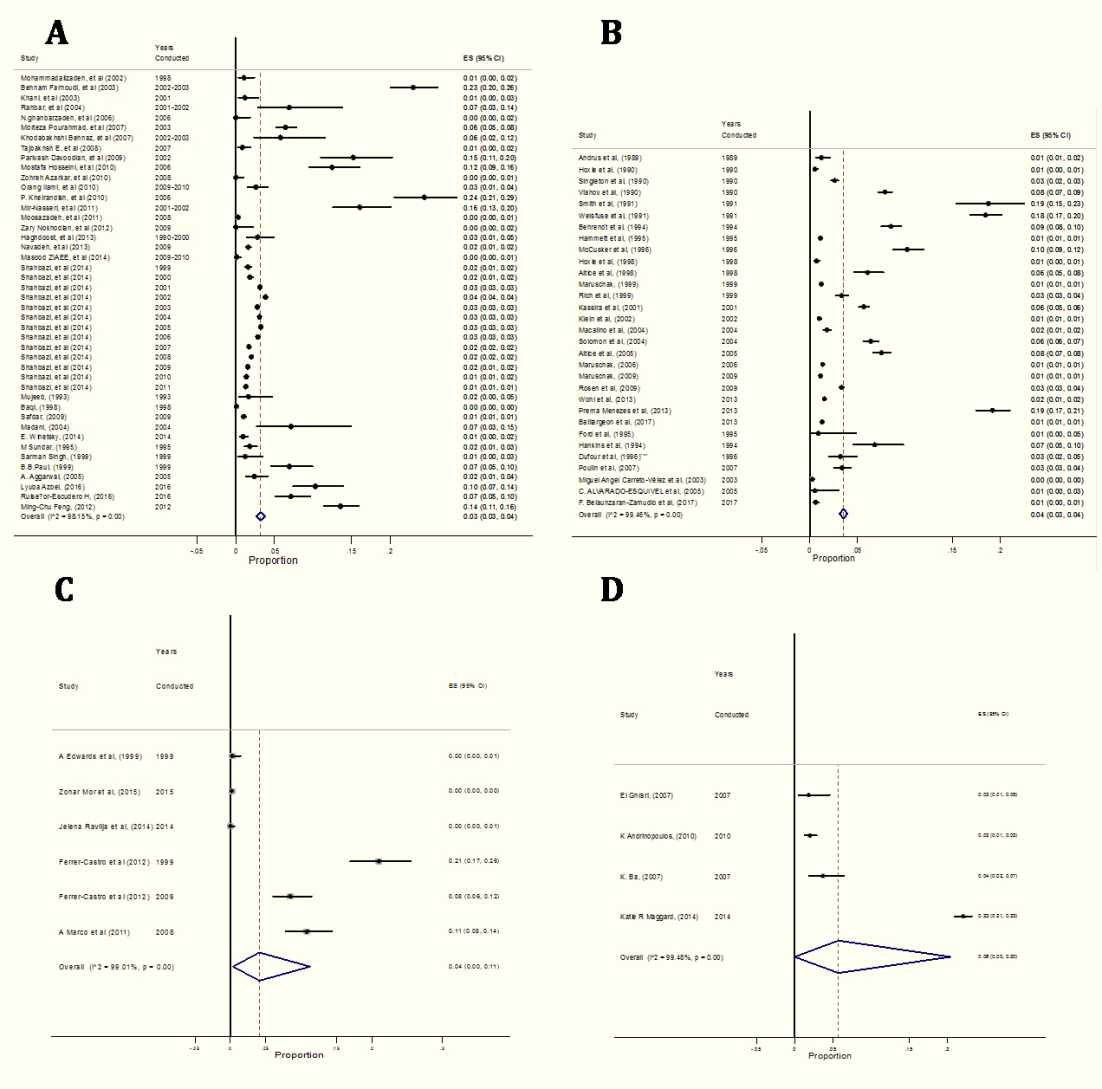
Forest plot for the overall prevalence of HIV among A. Asian prisoners B. North American prisoners C. European prisoners D. African prisoners. Each study is shown by the point estimate of the prevalence (p) and 95% confidence interval for the p (extending lines); the pooled p and 95% confidence interval by random-effects calculations are depicted as a diamond

The pooled estimate of HIV prevalence was 3.4% (95 % CI 3.2%–3.4%); however, the prevalence of HIV across individual studies varied considerably (ranging from 0 in Bosnia and Herzegovina to More than 20% in Zambia) and statistical heterogeneity was substantial (*I*^*2*^=0.99, Q=1; *P*<0.0001), Egger’s test revealed that there is significant publication bias (t=7.09; *P*<0.0001). The result of sensitivity analysis showed that pooled estimate of HIV prevalence was 2% (95% CI 2%–3%) (Table 1, Fig. 2).

**Table 1:** Country-based pooled analysis of the prevalence of HIV among prisoners

***Location***	***N***	***Heterogeneity test***		***I^2^***	***Egger’s Test***		***P (95% CI)***	***test(s) of P=0***	
		
		***Q Cochrane***	***P-value***		***Bias***	***P-value***		***Z***	***P-value***
Asia	32	2319.62	<0.0001	98.15%	9.28	0.79	0.03 (0.033, 0.043)	19.49	<0.0001
Africa	4	576.93	<0.0001	99.5%	7.23	<0.0001	0.06 (0.0, 0.20)	1.92	0.05
North America	31	5555.62	<0.0001	99.5%	5.88	0.19	0.04 (0.03, 0.04)	30.07	<0.0001
Europe	5	503.38	<0.0001	99%	7.39	<0.0001	0.05 (0.0, 0.11)	2.6	0.01
Overall	72	14613.19	<0.0001	99.43%	7.09	<0.0001	0.034 (0.032, 0.036)	33.34	<0.0001

Three Asian countries showed the HIV prevalence among prisoners greater than 10%, including Iran (15% in Southern region (24)), Kyrgyzstan (10.3%) (13), and Taiwan (13.5%) (29). Among North American countries, some region of USA showed the HIV prevalence among prisoners greater than 10% (18.75%) (73); while in Europe countries, three showed the HIV prevalence among prisoners greater than 10%, including Spain (21%) (30), Estonia (16%) (6), and Ukraine (14.5%) (6). The pooled prevalence of HIV among prisoners in the continents Asia, Africa, North America and Europe was estimated as 3.0% (95% CI 3.3%–4.3%), 6% (95% CI −0.0%–2.0%), 4% (95% CI 3.0%–4.0%), 5.0% (95% CI 0.0%–11%), respectively (Table 1, Fig. 2).

## Discussion

Although the discrimination and prejudice behavior in prisons in the world has exacerbated the spread of various diseases, including HIV/AIDS, among prisoners (2), few studies have been performed to document the prevalence of disease and associated behaviors among this population so far. This systematic review and meta-analysis included all available studies that reported on the prevalence of HIV among prisoners in the world.

HIV in many parts of the world is known more as a chronic disease than a lethal infectious disease (3). The HIV prevalence is different among various people, and both the number of CD4 cells and the viral load are useful in predicting outcomes (84). Without treatment, the survival time after HIV infection is estimated to be on average 9 to 11 yr, depending on the type of HIV (85). If the treatment begins after the diagnosis of HIV, life expectancy will be between 10 and 40 yr (86). HIV is a global epidemic (87). HIV is more likely to be transmitted*,* through unprotected sexual intercourse and syringes and needles used for injection (88). The needle sharing HIV infection occur in 69.5% and via unprotected sexually transmitted intercourse is 10% of the general population (89, 90). Africa, especially in the countries of sub-Saharan region is known as the first leading HIV burden part of the world (91).

Today’s, prisons are one of the well-known centers of focusing on HIV infection around the world, and not only prisoners are prone to HIV infection but they are considered as a reservoir for the onset and spread of HIV in the community. HIV-infection was presented in more than 10% of the general population in some African countries, including Botswana, Lesotho, Malawi, Mozambique, Namibia, South Africa, Swaziland, Zambia, and Zimbabwe (92). Our meta-analysis also showed a high HIV prevalence among prisoners in Africa. Although Asia that considers as the second major HIV burden, has important epidemiologic differences with Africa, the HIV incidence is declining in Asia as in sub-Saharan Africa. Unlike the high HIV prevalence in the general population and despite the high prevalence of AIDS in some regions of various Asian countries (93), our meta-analysis showed a low HIV prevalence among Asian prisoners.

## Conclusion

People in prisons have the same right to health and health care, including preventive measures, as those outside, and their lives and health are connected to those outside in many ways. Protecting prisoners’ health protects general public health. Successful HIV preventive measures in prisons include provision of HIV education and information; clean needles and syringes; drug treatment; and condoms. Governments have a moral and ethical obligation to prevent the spread of HIV/AIDS in prisons and to provide proper and compassionate care, treatment and support for those infected.

## Ethical considerations

Ethical issues (Including plagiarism, informed consent, misconduct, data fabrication and/or falsification, double publication and/or submission, redundancy, etc.) have been completely observed by the authors.
